# Toward a Deuterium Feather Isoscape for Sub-Saharan Africa: Progress, Challenges and the Path Ahead

**DOI:** 10.1371/journal.pone.0135938

**Published:** 2015-09-10

**Authors:** Carlos Gutiérrez-Expósito, Francisco Ramírez, Isabel Afán, Manuela G. Forero, Keith A. Hobson

**Affiliations:** 1 Departamento de Biología de la Conservación, Estación Biológica de Doñana (CSIC), Sevilla, Spain; 2 Laboratorio de SIG y Teledetección (LAST-EBD), Estación Biológica de Doñana (CSIC), Sevilla, Spain; 3 Environment Canada, Saskatoon, Saskatchewan, Canada; Union College, UNITED STATES

## Abstract

A key challenge to the application of continent-wide feather isoscapes for geographic assignment of migrant birds is the lack of ground-truthed samples. This is especially true for long-distance Palearctic-Afrotropical migrants. We used spatially-explicit information on the *δ* ^2^H composition of archived feathers from Green-backed/Grey-backed Camaroptera, to create a feather *δ* ^2^H isoscape for sub-Saharan Africa. We sampled from 34 out of 41 sub-Saharan countries, totaling 205 sampling localities. Feather samples were obtained from museum collections (n = 224, from 1950 to 2014) for *δ* ^2^H assay. Region, altitude, annual rainfall and seasonal patterns in precipitation were revealed as relevant explanatory variables for spatial patterns in feather *δ* ^2^H. Predicted feather *δ* ^2^H values ranged from -4.0 ‰ to -63.3 ‰, with higher values observed in the Great Rift Valley and South Africa, and lower values in central Africa. Our feather isoscape differed from that modelled previously using a precipitation *δ* ^2^H isoscape and an assumed feather-to-precipitation calibration, but the relatively low model goodness fit (F_10,213_ = 5.98, p<0.001, R^2^ = 0.18) suggests that other, non-controlled variables might be driving observed geographic patterns in feather *δ* ^2^H values. Additional ground-truthing studies are therefore recommended to improve the accuracy of the African feather *δ* ^2^H isoscape.

## Introduction

In order to manage or conserve migratory birds and other wildlife, it is essential to consider the complete annual cycle by making spatial connections between key breeding, migration and wintering sites [1]. While such migratory connections are poorly known for most of the world’s migratory birds, recent development of increasingly sophisticated and light weight tracking devices are moving the field forward at a rapid rate [2]. In particular, light-sensitive geologgers have been miniaturized to now be used effectively in studies of small (~15g) passerines but require recapture, and can have significant error especially during the equinox [3, 4]. Within this scenario, the use of intrinsic markers and particularly the analysis of stable isotopes in feathers has emerged as a powerful contribution to the ecologist’s toolbox by providing a relatively cheap method to describe migratory patterns and population connectivity [5]. Feathers are metabolically inert following formation and their isotopic composition thus reflects isotopic values derived from foodwebs at areas of growth. Isotopic values in foodwebs, in turn, can vary spatially in a predictable fashion creating isoscapes that can be a useful means of inferring origins of birds later sampled. So, providing feather moult is well understood, isotopic approaches can be used to assign feather isotopic signals to particular geographic areas [6, 7, 8, 9, 10].

To date, geographic assignments of birds to molt origins have relied extensively on measurement of stable-hydrogen isotope values (*δ* ^2^H) in feathers because they reflect amount-weighted long-term average *δ* ^2^H values in precipitation and these patterns are known reasonably well at the continental or global scale [11, 12, 13, 14]. By applying rescaling functions linking precipitation and feather *δ* ^2^H values [15], it is possible to model expected feather *δ* ^2^H isoscapes to be used in assigning individuals or populations to moult origins. However, for Africa, the distribution of precipitation stations that collect water for isotopic measurements under the Global Network for Isotopes in Precipitation (GNIP) are generally poor and it is also unclear if rescaling algorithms used to link precipitation and feather *δ* ^2^H derived for North American or European passerines can be applied to Africa [5, 16]. An alternate approach is to derive a feather *δ* ^2^H isoscape directly by collecting feathers from known origins across the continent [17, 13], but little direct information exists about the stable isotope distribution in feathers along the Afrotropical areas. Feather *δ* ^2^H isoscapes created using ground-truthed rescaling functions are therefore required to improve assignment accuracy in these areas [18, 19]. In Africa, widespread feather sampling is logistically extremely difficult but museum collections can be used as a source of feather samples at a continental scale. Here, we present, for the first time, a feather *δ* ^2^H isoscape for sub-Saharan Africa based on museum collections. Our motive was to investigate model parameters influencing feather *δ* ^2^H across Africa and to potentially provide an isoscape that could be used to assign European migratory birds to moult origins in Africa.

## Materials and Methods

### Sampling

**Fig 1:**
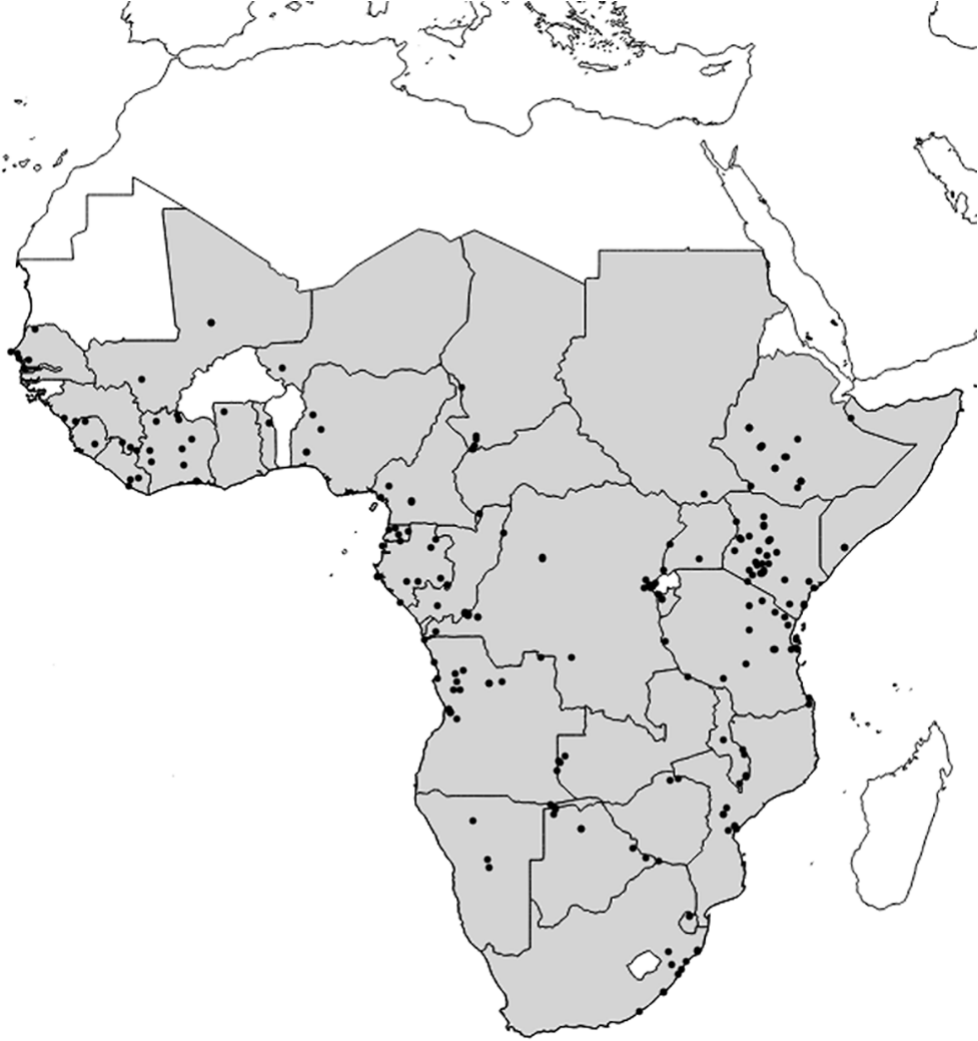
Sampling area. Shaded countries are those with at least one locality sampled, which are showed as black dots.

We concentrated our analyses on potential wintering areas in Africa of European-Afrotropical migrants and so excluded the Sahara desert and all the Mediterranean areas of the Maghreb, along with all African islands in both the Indian and the Atlantic Ocean. In order to avoid taxon-specific differences in the isotopic composition of feathers and to cover the whole sub-Saharan area of the continent we chose the Green-backed/Grey-backed Camaroptera complex (*Camaroptera brachyura/brevicaudata*, Vieillot, 1820), hereafter Camaroptera, as a surrogate species. This small bird is a common species and thus easily found in museum holdings; widely distributed throughout the African continent, allowing the use of a single species for the whole study area. The species is also largely sedentary [20], so that we can assume that any feather analyzed was grown at the collection site. Finally, the species is mostly insectivorous and so is consistent with diets expected for most European long-distance Passerines that winter and moult in Africa. We analyzed 224 samples from museum specimens of known locations (< 10 Km accuracy), from 205 localities distributed along 34 sub-Saharan countries (Fig 1). Most specimens were on loan from American Museum of Natural History (New York, USA), Carnegie Museum of Natural History (Pittsburgh, USA), Doñana Biological Station (Sevilla, Spain), Delaware Museum of Natural History (Wilmington, USA), Field Museum (Chicago, USA), Kansas University Museum of Natural History (Lawrence, USA), Muséum National d’Histoire Naturelle (Paris, France), Naturalis Biodiversity Center (Leiden, The Netherlands), Natural History Museum of Denmark (Copenhagen, Denmark), Western Foundation for Vertebrate Zoology (Camarillo, USA), Yale Peabody Museum of Natural History (New Haven, USA), plus two samples obtained during field expeditions by Muséum National d’Histoire Naturelle staff in Guinea Conakry, seven samples obtained by the Research Centre in Biodiversity and Genetic Resources (CIBIO, Portugal) under the collaboration protocol with the Zoological Museum and Herbarium of Lubango (ISCED-Huíla, Angola) and one found as a road kill in Windhoek (Namibia) (S1 Table—Accession numbers of museum specimens used in this study).

### Isotope analysis

Feathers were cleaned with ethanol, rinsed with ultrapure water (Milli-Q from Millipore Corporation), dried in clean open vials at 45°C in a drying oven and before weighting, keratin standards (CBS, KHS and LIE-PA2) and samples are equilibrated together with lab air for at least 5 days prior to isotopic analysis in order to avoid effects of H exchange with ambient water vapor. Subsamples were weighed into silver capsules and analyzed at the Stable Isotope Laboratory at Doñana Biological Station (LIE-EBD) using a Flash HT Plus Elemental Analyzer coupled to a Delta-V Advantage Isotope Ratio Mass Spectrometer (IRMS) via a Conflo IV interface (Thermo Fisher Scientific, Bremen, Germany). A comparative equilibration approach was used based on Wassenaar and Hobson [21]; standards used were CBS (Caribou hoof: -197 ‰) and KHS (Kudu horn: -54.1 ‰) from Environment Canada and LIE-PA2 (Razorbill feathers: 20.8 ‰) from Doñana Biological Station. Based on replicate within-run measurements of standards, we estimate measurement error to be ±3‰. All *δ* ^2^H values are reported relative to the Vienna Standard Mean Ocean Water (VSMOW) scale.

### Model construction

We first compared our measured feather *δ* ^2^H values with those predicted from the theoretical African feather isoscape raster map created by Hobson et al. [14]. That isoscape was based on an amount-weighted mean annual grid map of *δ* ^2^H values in precipitation at a 16.5 km spatial resolution as sourced online primarily from the Global Network of Isotopes in Precipitation (GNIP) database from IAEA/WMO (http://www-naweb.iaea.org) [22]. In a second step, values from this database were also used to examine whether the observed variability between measured and predicted feather *δ* ^2^H values could be explained by the effect of phenological differences in the moult period for the different Camaroptera populations sampled. Finally, we examined if additional covariates other than latitude and altitude [23] may have contributed to the observed variability in feather *δ* ^2^H values. We extracted all environmental variables associated with each sampling location that could have an important role in influencing precipitation *δ* ^2^H values [11]. Monthly precipitation (mm) rasters were obtained from WorldClim [24] (http://www.worldclim.org) at 1 km spatial resolution. Altitude, was extracted from the GLOBE Project at 1 km spatial resolution [25] (http://www.ngdc.noaa.gov/mgg/topo/globe.html), and compared with data provided by specimen label when altitude was available, in the very few cases of discordance, we used label data.

One of the challenges with creating a feather isoscape is determining over which period precipitation should be averaged to reflect the major H signal in the foodweb leading to birds at the time of moult [26]. Vegetation growth is highly correlated with the photosynthetically active radiation absorbed by the plant canopy, and thus with the Normalized Difference Vegetation Index (NDVI), accounting for the study of phenological changes in vegetation [27] and thus deduce the most likely moulting season for each considered pixel. Mean and standard deviation of monthly NDVI (extracted from satellite NOAA-AVHRR images) computed over an 18-year period (from 1982 to 2000, excluding 1994 data from calculation) were downloaded from EDIT Geoplatform at 10 km spatial resolution [28] (http://edit.csic.es/Soil-Vegetation-LandCover.html). Global evapotranspiration from the MODIS sensor (MOD16 product, 1 km spatial resolution) was downloaded from the University of Montana [29], ftp.ntsg.umt.edu. Monthly mean values for the 2000–2010 time period were used.

From monthly precipitation data, we reconstructed intra-annual rainfall patterns for each sample location. Assuming that reproduction takes place during the rainy season, and that moult is performed mostly after breeding [30, 31, 32], we assigned a moulting period for each sampled bird based on seasonal patterns. We assigned the most likely month for moulting body feathers as two months after the maximum rain peak (hereafter *moult month*). We also considered the moulting period as the four months after the maximum rain peak (hereafter *moult season*) and before the beginning of the dry season. Predicted precipitation *δ* ^2^H values for moult month and moult period were weighted by the monthly rain amount. Regionalized cluster-based water isotope prediction (RCWIP) from the International Atomic Energy Agency [33, 34] was used to group feather samples into six climatic cluster domains, hereafter regions. The RCWIP allowed us to assign to all pixels in the study area a relative weight of belonging to each of the considered regions (S1 Fig—African regionalization by using climatic regression models). Because rain is the main source of water *δ* ^2^H, monsoonal and Atlantic fronts undoubtedly play an important role in the distribution pattern of precipitation *δ* ^2^H values across the continent. Thus, minimum distance from each sampled locality to the coast was also included as a covariate. Finally, we calculated a rain seasonality index following Walsh & Lawler [35] with values under 0.19 denoting precipitation spread throughout the year, and with values over 1.2 indicating extreme seasonality with almost all precipitation occurring in one or two months.

We considered up to seven explanatory variables: absolute latitude (ABS_LAT), annual precipitation (PREC-TOT), distance to the coast (DIST_SEA), Walsh Lawler seasonality index (SI), altitude (ALT), Normalized Difference Vegetation Index (NDVI) and Evapotranspiration (ET); bioclimatic region (REGION), with six levels [34] as a factor. Correlations among continuous variables were explored through a Pearson correlation matrix. Both NDVI and ET showed a high correlation (Pearson’s r = 0.62), and were also significantly correlated with PREC_TOT (Person’s r = 0.434 and r = 0.57, respectively). Additionally, DIST_SEA was highly correlated with ALT (Pearson’s r = 0.54). Accordingly, these three variables were excluded from further analyses. A set of competing models was built by considering: PREC_TOT, SI, ALT as well as ABS_LAT and its quadratic term, REGION as factor. PREC_TOT was selected instead of precipitation during *moult month* or *moult season*, since our feather *δ* ^2^H values showed a better correlation with annual-averaged rainfall *δ* ^2^H values than period-specific rainfall *δ* ^2^H values (see results). Model selection was done using the Akaike information criteria corrected for small sample sizes (AIC_C_) and the corresponding AIC_C_ increments (ΔAIC_C_ < 2) and weights (AIC_C_ Wgt). Geographical information were treated by using ArcGis 10.0 software (ESRI, Redland, USA), whereas statistical analyses were performed using R statistical environment 3.0.2., and the *MuMIn* [36], *lme4* [37] and *arm* [38] packages.

## Results

**Fig 2:**
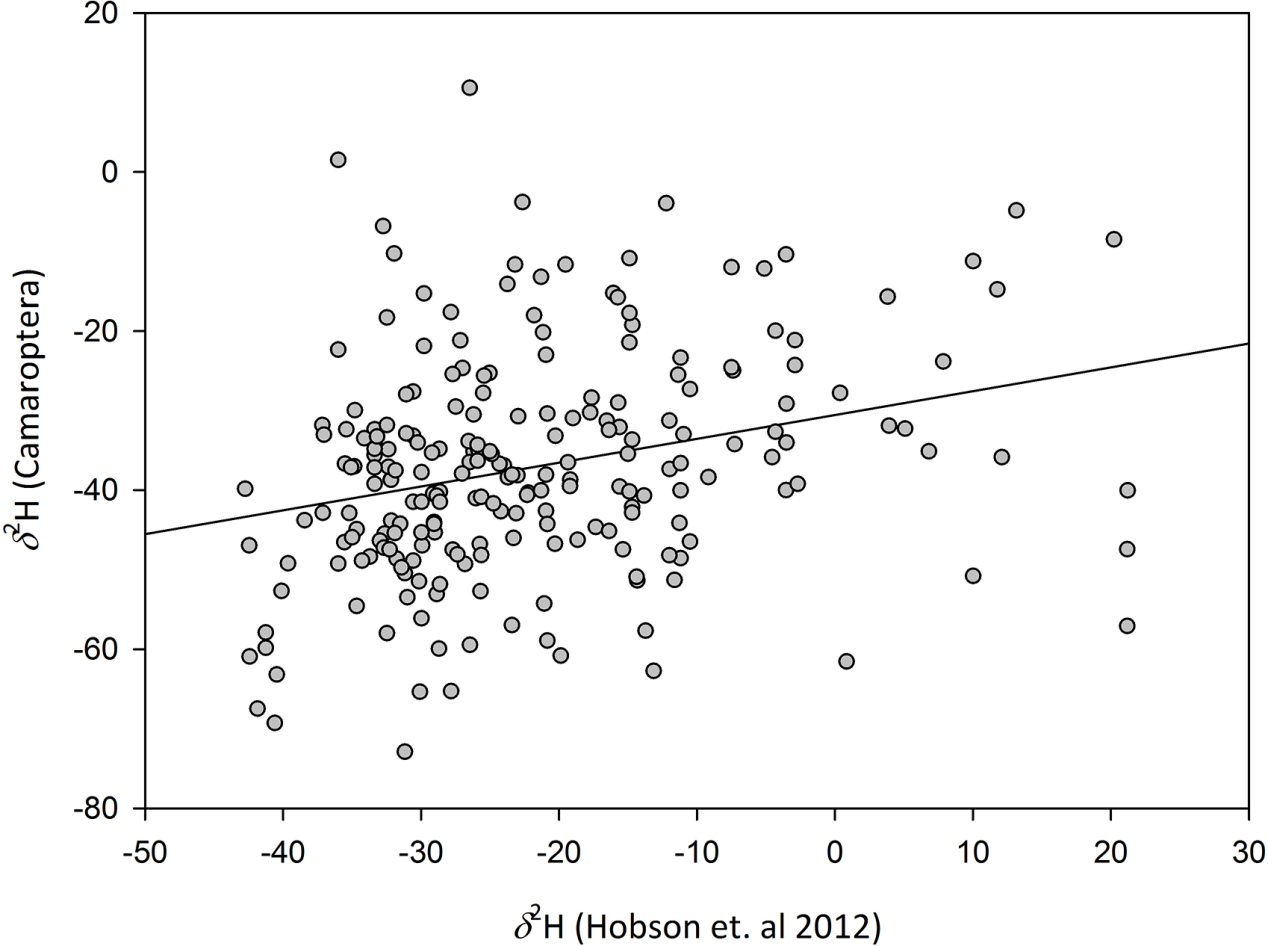
Dispersion plot and regression of Camaroptera *δ* ^2^H versus deuterium values from Hobson et al. 2012 [14].

Linear regression between estimates of feather *δ* ^2^H based on Hobson et al. [14] and our Camaroptera feather *δ* ^2^H results yielded a significant relationship but with a relatively low explanatory power (F_1,222_ = 16.68, p<0.001, R^2^ = 0.07) (Fig 2). The same analysis was done comparing *δ* ^2^H in Camaroptera feathers with mean annual precipitation *δ* ^2^H values (F_1,222_ = 25.71, p<0.001, R^2^ = 0.09) and those for the *moult month* (F_1,222_ = 4.16, p = 0.04, R^2^ = 0.02) and the *moult season* (F_1,222_ = 12.9, p<0.001, R^2^ = 0.05). Higher correlation was obtained when relating Camaroptera feather *δ* ^2^H values against total precipitation *δ* ^2^H values than those based on moulting periods and so we considered only annual rainfall as covariate.

Following Bowen & Revenaugh [23] and Bowen [39], we made a Linear Model, using as covariates altitude, absolute latitude and lat^2^. Although both altitude and latitude affected *δ* ^2^H in Camaroptera feathers, the model was not significant (F_3,220_ = 3.54, p = 0.015, R^2^ = 0.03).

**Fig 3:**
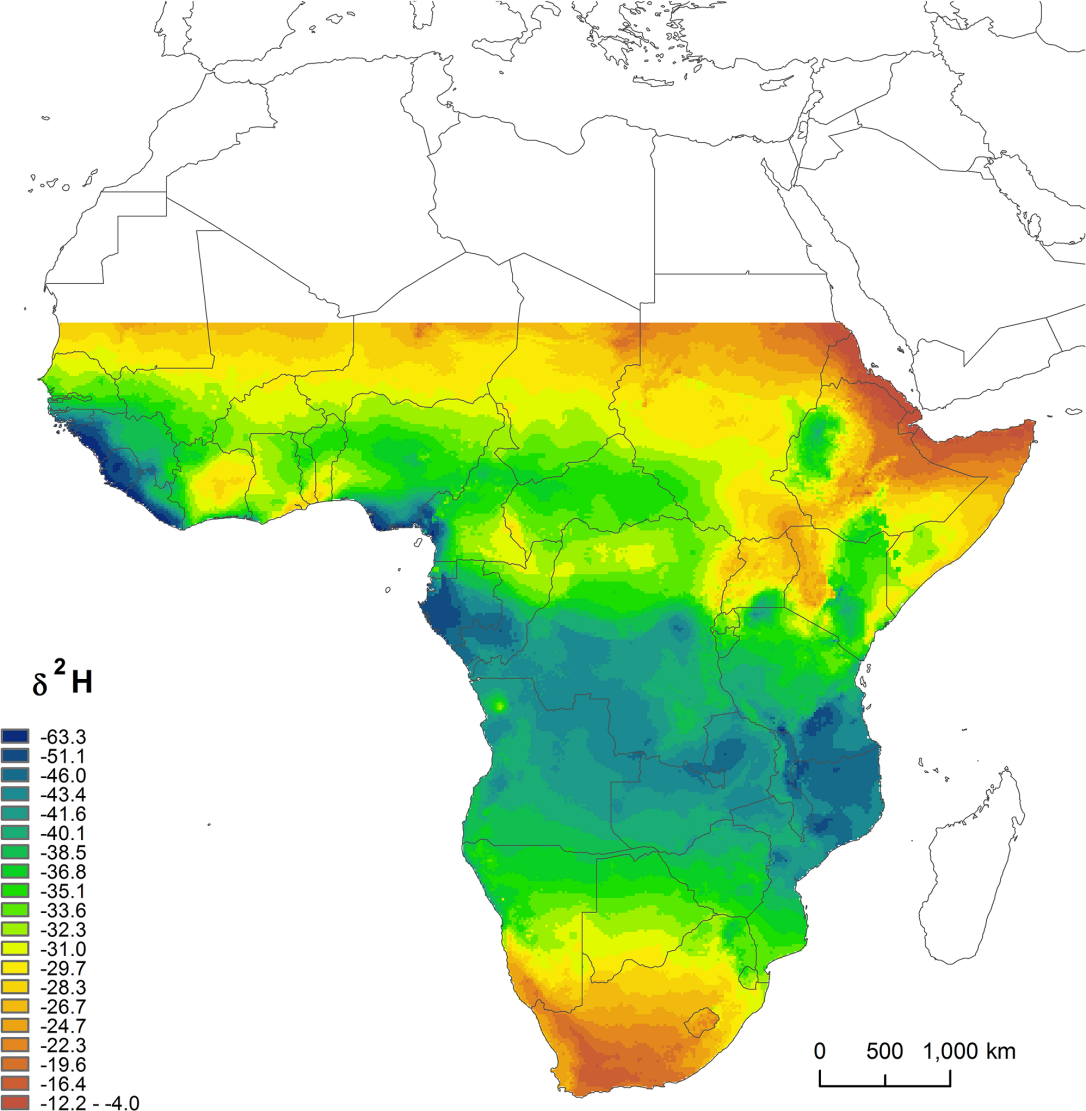
Camaroptera feather *δ* ^2^H isoscape resulting from the application of the model obtained.

Our top ranked model included all variables and had a weight of 0.73. That Linear Model was highly significant (F_10,213_ = 5.98, p<0.001, R^2^ = 0.18). Covariates coefficients, confidence intervals and relative importance of covariates of this model are shown in Table 1 and were used, together with the covariates grids and probability of each pixel to belong to a region, to build a Camaroptera feather isoscape. The resulting map is shown in Fig 3.

**Table 1:** Selected model coefficients, confidence interval and relative importance of the variables used.

	Estimate	Std. Error	t value	Pr(>|t|)
(Intercept)	4.785867	13.429	0.36	0.722
ABS_LAT^2^	0.024396	0.019	1.30	0.200
ABS_LAT	-0.481181	0.549	-0.88	0.382
ALT	0.003328	0.001	2.25	0.025
PREC_TOT	-0.008978	0.002	-4.78	< 0.001
SI	-14.578157	6.076	-2.40	0.017
RegV11	-14.103769	13.260	-1.06	0.289
RegV13	-25.349481	12.900	-1.97	0.051
RegV14	-17.133694	13.092	-1.31	0.192
RegV15	-27.852456	13.446	-2.07	0.040
RegV26	-25.3549	14.320	-1.77	0.078

**Fig 4:**
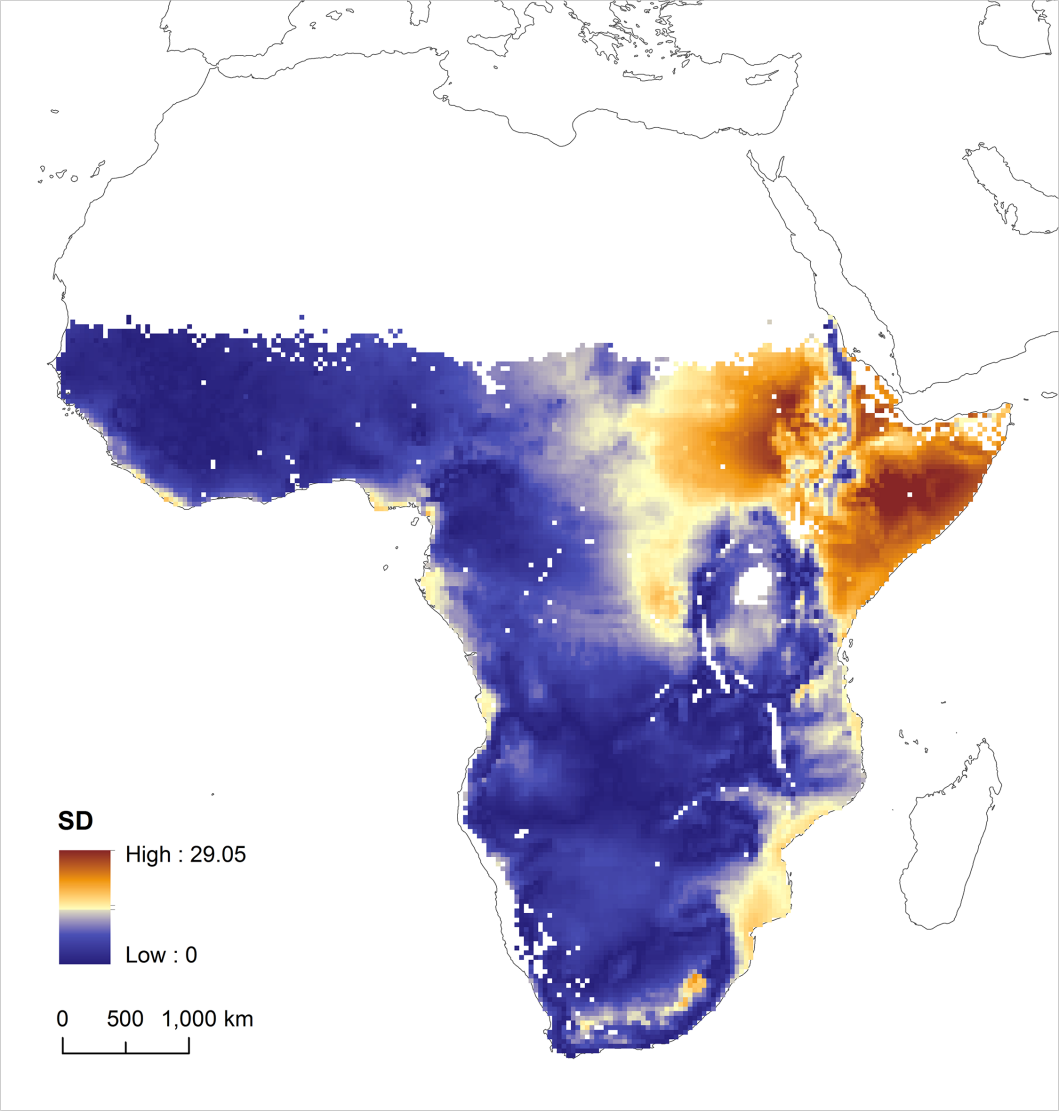
Standard deviation map resulting from the comparison between the Camaroptera feather *δ* ^2^H isoscape (present work) and the feather *δ* ^2^H isoscape predicted by Hobson et al. (2012) [14].

In order to compare our results with those predicted by Hobson et al [14], we resampled our high spatial resolution *δ* ^2^H feather map to match the spatial resolution of previously published feather isoscapes (0.33 degrees cell size). Once both maps were resampled, we calculated the standard deviation of both values for each pixel in order to explore the spatial distribution of mismatches (Fig 4).

## Discussion

We provide for the first time, a high resolution ground-truthed feather *δ* ^2^H isoscape for African insectivorous passerines based on a model that considered latitude, altitude, annual precipitation, a seasonality index and geographical region as covariates. We expected a better correlation among our Camaroptera feather *δ* ^2^H values and the theoretical *δ* ^2^H isoscapes model derived by Hobson et al. [14] but high dispersion in our data suggested that we were unable to control for all influential variables and greater refinement of our model is now needed. We suspect that variation in timing of moult for Camaroptera and uncertainties and assumptions inherent in museum collections of feathers collected over many decades, together with inherent complexities in modeling precipitation and foodweb *δ* ^2^H for Africa, all contributed to total model variance. However, and owing to the low signal to noise ratio in the isotopic composition of feathers, the small isotopic variance of the whole suite of samples with respect expected isotopic variance based on individuals of known moult origin [39], our model could be still doing a reasonable job of describing the component of that variance attributable to large-scale environmental factors.

Differences among the predicted mean annual precipitation *δ* ^2^H values and those for actual mean *δ* ^2^H in precipitation contributing H to the foodweb leading to Camaroptera feathers is likely a primary source of error in our feather isoscape. Values of *δ* ^2^H in meteoric water can vary greatly from the rainy season through the dry period, with enrichment of water available to plants also due to evapotranspiration [40]. In many regions of Africa, we suspect that local foodweb *δ* ^2^H can differ considerably from that predicted by models that assume periods of plant water integration leading to birds. Unfortunately, knowledge about timing of moult in Afrotropical birds is poor with few studies [41, 42, 43] and only a few researchers have examined Camaroptera in particular [30, 32, 31]. We predicted a *moult season* and a *moult month* for the sampled Camaropteras, but narrowing periods of H integration into the foodweb for these periods generally resulted in poorer model fit than those based on annual rainfall. This may be due to the assumption that Camaropteras only moult after the breeding season and that reproduction takes place at the beginning of the rainy season. Although this must be true in broad sense, local adaptations to more than one breeding season or to different habitats within the same site, a greater mismatch between rains and reproduction and thus moult process, all can occur and indeed an unexpected variation in deuterium values within the same locality [44]. Stations in Africa contributing to the GNIP database are also relatively few (20 are found within the study area boundaries) [23] and so a more refined precipitation *δ* ^2^H basemap is clearly needed for this continent. Similarly, the rescaling function used by Hobson et al. [14] to transform the African precipitation *δ* ^2^H isoscapes into a *δ* ^2^H feather isoscape was based on a calibration using known-origin Eurasian Reed Warbler (*Acrocephalus scirpaceus*) in Europe [16] and we do not know how well this function applies to African-grown Camaroptera feathers.

Most differences between the feather *δ* ^2^H values predicted by Hobson et al. [14] and those predicted by our Camaroptera model were found in the northeast of our study area, corresponding mostly to the Ethiopian highlands, north and south of the Great Rift Valley and the Horn of Africa. Here, expected feather *δ* ^2^H values were much more positive than those obtained in the Camaroptera sampled by us. Camaroptera sampled in Sudan, Ethiopia and Somalia had feather *δ* ^2^H values ranging between -61.6‰ and 1.5‰, while the predicted values from Hobson et al. [14] were all positive. This region has a complex isotope hydrology. Precipitation patterns are dominated by monsoonal seasonality, where most of the rains occur at high altitude (i.e. Bale Mountains) and so are expected to be less enriched in ^2^H compared with precipitation in the dryer and lower surrounding areas. Foodwebs can also be fed by rivers flowing from the mountains to the Nile basin and to the Indian Ocean lowlands. Camaropteras may moult in cooler or shaded habitats with corresponding foodweb *δ* ^2^H values lower than those based solely on larger scale averages. Although the only positive feather *δ* ^2^H value (+ 1.5 ‰) we measured for the whole continent was obtained in Ethiopia, the *δ* ^2^H predicted value for that location was much more positive (i.e. 35.8‰)

Our study indicates that much more on-the-ground sampling and isotopic measurement of feathers of known moult origin are required for Africa. Efforts to increase our ability to predict rainfall *δ* ^2^H through an increase in GNIP stations will also be crucial together with refinement of our understanding of moult in targeted species. Africa is a complex continent isotopically and much effort will be required to improve our feather isoscapes to permit key studies on migratory connectivity between African wintering grounds and European breeding grounds for a variety of species. Nonetheless, previous studies in Africa and North America indicate that the rewards for this effort can be considerable [14, 45, 46]. We especially encourage those researchers engaged in the use of light-sensitive geolocators and/or gps tags [47] to track Eurasian-Afrotropical migrants to sample feathers of birds of known trajectory in order to assist in elucidating patterns of feather isotopes for Africa.

## Supporting Information

S1 Fig African regionalization using climatic regression models, obtained from Terzer et al. [34]. (TIF)

S1 Table Accession number of the specimens used on this study, with reference to coordinates, location and country of origin as well as scientific collection origin. Raw *δ* ^2^H values are included. (DOCX)
