# Supplementary figures and images for: Toward a Deuterium Feather Isoscape for Sub-Saharan Africa: Progress, Challenges and the Path Ahead
Source: PLoS One. 2015 Sep 10;10(9):e0135938. doi: 10.1371/journal.pone.0135938 (PMC4565548; doi:10.1371/journal.pone.0135938)

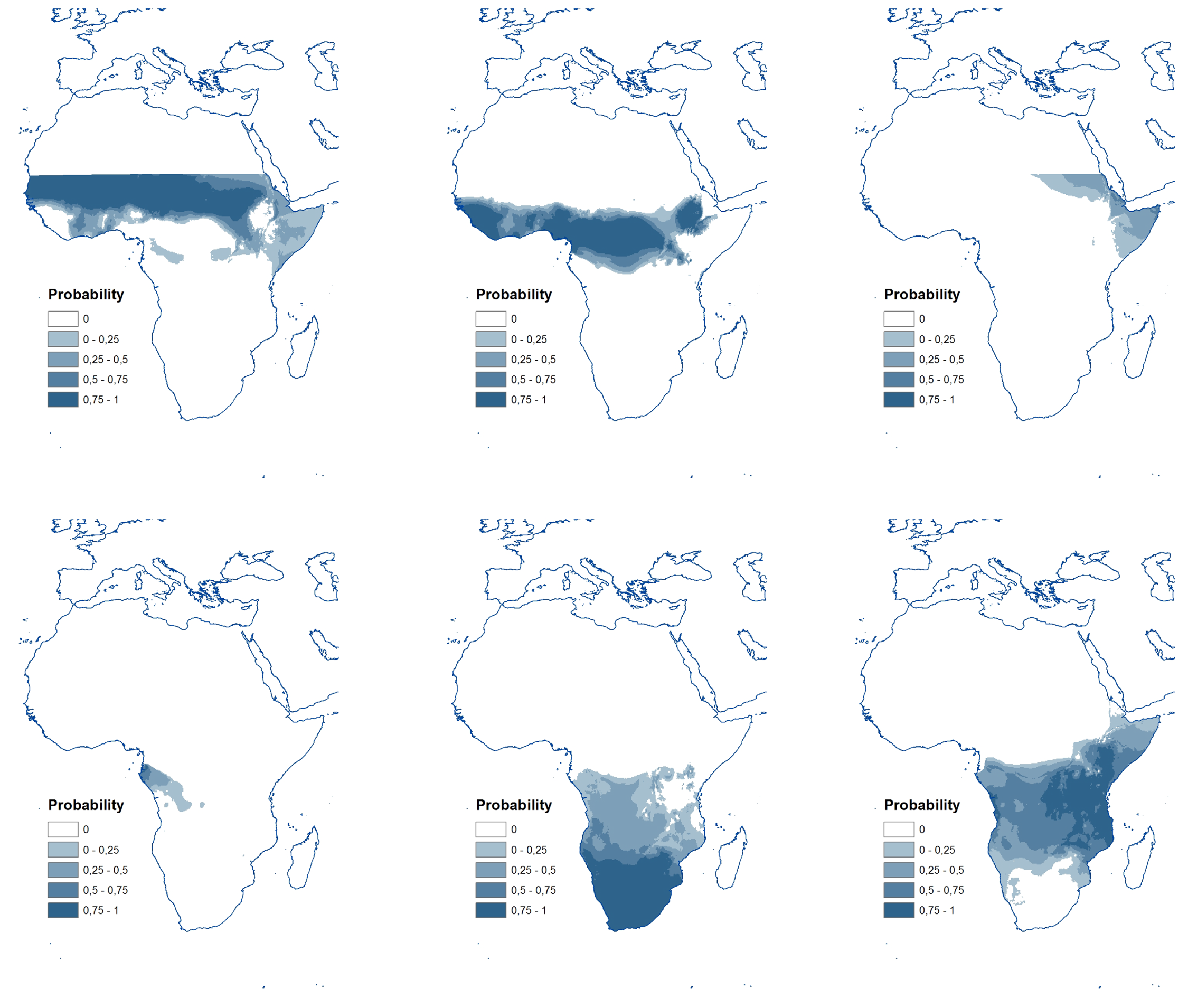

Supplement: S1 Fig — (TIF) [file pone.0135938.s001.tif]
