# Supplementary material for: Toward a Deuterium Feather Isoscape for Sub-Saharan Africa: Progress, Challenges and the Path Ahead
Source: PLoS One. 2015 Sep 10;10(9):e0135938. doi: 10.1371/journal.pone.0135938 (PMC4565548; doi:10.1371/journal.pone.0135938)
Supplement: S1 Table — (DOCX) [file pone.0135938.s002.docx]

| **Country** | **Museum** | **Accesion number** | **Latitude** | **Longitude** | **Location** | ***δ*^2^H values** |
| --- | --- | --- | --- | --- | --- | --- |
| Angola | CIBIO | W38509 | -11.36 | 14.20 | Bango-Seles | -41.49 |
| Angola | CIBIO | W38515 | -11.36 | 14.20 | Bango-Seles | -37.75 |
| Angola | CIBIO | W38516 | -11.36 | 14.20 | Bango-Seles | -56.13 |
| Angola | CIBIO | W38504 | -11.12 | 14.18 | Bimbe | -45.32 |
| Angola | FM Chicago | FM221069 | -8.30 | 15.20 | Canzele 30 km west of Camabatela | -46.57 |
| Angola | FM Chicago | FM250648 | -9.15 | 18.04 | Cuango | -43.86 |
| Angola | YPM Yale | YPM ORN 095528 | -7.38 | 20.83 | Dundo | -38.73 |
| Angola | FM Chicago | FM216581 | -9.13 | 14.77 | Golungo Alto | -46.04 |
| Angola | CIBIO | AF80104 | -11.18 | 14.10 | Jombe | -62.73 |
| Angola | FM Chicago | FM221070 | -8.96 | 13.35 | 17 km ESE of Luanda | -39.56 |
| Angola | FM Chicago | FM221059 | -9.22 | 17.06 | Luhanda. 5 km N Quela | -32.10 |
| Angola | FM Chicago | FM221056 | -9.22 | 17.06 | Luhanda. 5 km N Quela | -40.46 |
| Angola | FM Chicago | FM250649 | -7.74 | 13.12 | Maladi | -40.04 |
| Angola | FM Chicago | FM225009 | -9.67 | 14.44 | Mucoso. near Dondo | -46.76 |
| Angola | CIBIO | AF80194 | -11.84 | 14.76 | Namba/Forest Camp 1 | -46.97 |
| Angola | CIBIO | A-MM12 | -8.57 | 14.60 | Quibaxe | -37.09 |
| Angola | YPM Yale | YPM ORN 095532 | -9.75 | 14.93 | Quitando. 25 kilometes n of Calulo | -41.04 |
| Angola | FM Chicago | FM250650 | -6.14 | 12.35 | Santo Antonio do Zaire | -38.70 |
| Botswana | KU Lawrence | KU120356 | -21.83 | 28.41 | Central District. Lepokole Hills. 16 km N of Bobonong | -11.64 |
| Botswana | MNHN Paris | MNHN19692010 | -21.17 | 27.50 | Francis town | -35.13 |
| Botswana | MNHN Paris | MNHN19692006 | -22.14 | 29.30 | lower Shashi river | -21.44 |
| Botswana | FM Chicago | FM277547 | -18.28 | 21.79 | Mohembo | -58.01 |
| Botswana | FM Chicago | FM263483 | -18.36 | 21.84 | Shakawe | -47.24 |
| Botswana | FM Chicago | FM269227 | -19.76 | 23.67 | Shorobe | -18.32 |
| Botswana | FM Chicago | FM269226 | -19.76 | 23.67 | Shorobe | -37.92 |
| Botswana | FM Chicago | FM263485 | -18.75 | 21.75 | Tsodilo | -53.48 |
| Burundi | FM Chicago | FM358089 | -2.88 | 29.32 | Bukinanyama. Giserama. Kibira National Park | -42.92 |
| Burundi | FM Chicago | FM350873 | -3.18 | 29.57 | Mt Teza. Kibira National Park | -32.87 |
| Burundi | FM Chicago | FM346480 | -3.19 | 29.54 | Teza. 2.3 km N. .7 km W. Kibira National Park | -27.96 |
| Cameroon | AMNH New York | AMNH809738 | 4.16 | 9.23 | Great Sappo. Buea | -53.10 |
| Cameroon | MNHN Paris | MNHN19941021 | 5.03 | 9.83 | Mt Manengouba | -49.24 |
| Cameroon | MNHN Paris | MNHN19941029 | 3.87 | 11.45 | Nkolbisson | -48.64 |
| Cameroon | MNHN Paris | MNHN19941023 | 3.94 | 11.49 | Yaoundé Mt Fébé | -40.73 |
| Central African Republic | FM Chicago | FM429605 | 3.03 | 16.40 | Parc National de Dzanga-Ndoki. Mabea Bai | -25.28 |
| Democratic Republic of Congo | FM Chicago | FM313635 | -4.45 | 16.20 | Bankana | -58.94 |
| Democratic Republic of Congo | FM Chicago | FM318456 | -4.45 | 16.20 | Bankana | -45.48 |
| Democratic Republic of Congo | AMNH New York | AMNH764501 | -2.12 | 28.63 | Bataillon. nr. Bunyakiri. Kivu | -32.36 |
| Democratic Republic of Congo | KU Lawrence | KU121070 | -0.24 | 20.88 | Boende. 4.2 km north | -30.39 |
| Democratic Republic of Congo | FM Chicago | FM490097 | -0.17 | 20.93 | ca. 14 km N Boende | -44.28 |
| Democratic Republic of Congo | FM Chicago | FM429778 | -2.24 | 28.81 | Centre de Recherche N.S.. Lwiro | -15.30 |
| Democratic Republic of Congo | FM Chicago | FM434578 | -2.24 | 28.81 | Centre de Recherche N.S.. Lwiro. 3 mi E | -37.18 |
| Democratic Republic of Congo | FM Chicago | FM213460 | -2.18 | 14.03 | Djambala. Bateke Plateau | -48.37 |
| Democratic Republic of Congo | FM Chicago | FM429784 | -1.99 | 29.06 | Idjwi Island. Washiha Forest | -21.90 |
| Democratic Republic of Congo | FM Chicago | FM213461 | 1.63 | 18.05 | Impfondo. Oubangui River | -40.61 |
| Democratic Republic of Congo | AMNH New York | AMNH764502 | -1.83 | 28.43 | Irangi. Luhoho R. | -30.24 |
| Democratic Republic of Congo | FM Chicago | FM443912 | -2.40 | 28.23 | Kahuzi-Biega National Park | -31.31 |
| Democratic Republic of Congo | FM Chicago | FM443911 | -2.26 | 28.76 | Karaherere Swamp. Kahuzi-Biega National Park | -35.58 |
| Democratic Republic of Congo | MNHN Paris | MNHN19761426 | -4.20 | 15.55 | Kimpoko | -44.94 |
| Democratic Republic of Congo | FM Chicago | FM318450 | -4.20 | 15.55 | Kimpoko | -41.50 |
| Democratic Republic of Congo | FM Chicago | FM318448 | -4.33 | 15.52 | Kinkole | -33.18 |
| Democratic Republic of Congo | KU Lawrence | KU121007 | -5.57 | 13.16 | Luki Biosphere Reserve | -40.87 |
| Democratic Republic of Congo | FM Chicago | FM318451 | -4.36 | 15.48 | Mikonga | -27.63 |
| Democratic Republic of Congo | FM Chicago | FM434582 | -2.22 | 28.86 | Mugeri Seminary | -34.83 |
| Democratic Republic of Congo | FM Chicago | FM318447 | -7.44 | 23.02 | Nisele | -46.36 |
| Democratic Republic of Congo | AMNH New York | AMNH764499 | -2.22 | 28.78 | Tshibati. nr. Lwiro. Kivu | -34.31 |
| Democratic Republic of Congo | FM Chicago | FM438832 | -2.31 | 28.74 | Tshivanga | -39.22 |
| Equatorial Guinea | EBD Sevilla | EBD12360A | 1.90 | 10.26 | Alosa | -48.18 |
| Equatorial Guinea | EBD Sevilla | EBD12357A | 1.86 | 9.77 | Asonga | -56.98 |
| Equatorial Guinea | EBD Sevilla | EBD12389A | 1.03 | 10.66 | Ayasong. Acurenam | -38.70 |
| Equatorial Guinea | EBD Sevilla | EBD13026A | 1.71 | 11.18 | Mongomo | -31.83 |
| Equatorial Guinea | EBD Sevilla | EBD14622A | 1.48 | 10.53 | Yengue | -37.54 |
| Ethiopia | MNHN Paris | MNHN19981266 | 7.84 | 36.66 | 10km d'Agaro vers Jimma | -34.05 |
| Ethiopia | MNHN Paris | MNHN19981265 | 7.84 | 36.66 | 10km d'Agaro vers Jimma | -40.18 |
| Ethiopia | MNHN Paris | MNHN19780428 | 5.04 | 36.02 | 30km au nord de Kalam. SW | -4.85 |
| Ethiopia | MNHN Paris | MNHN19981273 | 7.92 | 36.80 | 38 km au nord de Jimma. vers suntu | -61.55 |
| Ethiopia | MNHN Paris | MNHN19981267 | 8.40 | 39.39 | 3km de Sodere | -14.80 |
| Ethiopia | MNHN Paris | MNHN19981271 | 9.18 | 35.87 | 5km à l'est de Gimbi | 1.50 |
| Ethiopia | MNHN Paris | MNHN19981268 | 9.18 | 35.87 | 5km à l'est de Gimbi | -15.68 |
| Ethiopia | MNHN Paris | MNHN19981274 | 4.90 | 39.36 | 68Km de Neghelli vers Watchile | -11.24 |
| Ethiopia | MNHN Paris | MNHN19981270 | 9.18 | 35.87 | 8km à l'est de Gimbi | -22.35 |
| Ethiopia | MNHN Paris | MNHN19981272 | 5.35 | 39.56 | 8km au nord de Neghelli | -29.14 |
| Ethiopia | MNHN Paris | MNHN20071022 | 7.06 | 38.48 | Awassa | -27.80 |
| Ethiopia | MNHN Paris | MNHN19981264 | 7.04 | 38.38 | Lac Awassa rive ouest | -31.93 |
| Ethiopia | MNHN Paris | MNHN19981256 | 6.25 | 37.77 | Lac Marguerite rive ouest | -12.16 |
| Ethiopia | MNHN Paris | MNHN19981258 | 6.25 | 37.77 | Lac Marguerite rive ouest | -10.86 |
| Ethiopia | MNHN Paris | MNHN19981262 | 5.35 | 39.56 | Neghelli | -17.74 |
| Ethiopia | MNHN Paris | MNHN19981261 | 5.35 | 39.56 | Neghelli | -23.85 |
| Gaboon | MNHN Paris | MNHN19830690 | 1.15 | 13.20 | Bélinga | -51.49 |
| Gaboon | FM Chicago | FM210424 | -1.55 | 9.04 | Fernan Vaz | -57.10 |
| Gaboon | FM Chicago | FM210425 | -1.55 | 9.04 | Fernan Vaz | -60.82 |
| Gaboon | EBD Sevilla | EBD20595A | -1.63 | 13.58 | Franceville | -46.95 |
| Gaboon | FM Chicago | FM210422 | 0.61 | 9.32 | Libreville. Cap Esterias | -47.44 |
| Gaboon | MNHN Paris | MNHN19830688 | 0.56 | 12.86 | Makokou. Ogooue. Ivindo | -44.26 |
| Gaboon | MNHN Paris | MNHN19540057 | -3.43 | 10.65 | Mayumba | -54.28 |
| Gaboon | FM Chicago | FM210727 | -1.89 | 11.92 | Mbigou. Mt du Chaillu | -45.34 |
| Gaboon | FM Chicago | FM213089 | -1.86 | 11.06 | Mouila. Mt Tandou | -36.92 |
| Gambia | WFVZ Camarillo | WFVZ29549 | 13.46 | -16.61 | Banjul. Fajara | -33.28 |
| Ghana | KU Lawrence | KU110868 | 10.40 | -2.06 | Gbele Resource Reserve | -59.94 |
| Guinea Conakry | MNHN Paris | 3549 | 9.89 | -13.63 | Dhoti 1 | -42.91 |
| Guinea Conakry | NHMD | 72984 | 7.75 | -8.82 | N'zérékoré | -33.51 |
| Guinea Conakry | MNHN Paris | 3712 | 9.69 | -12.82 | Yorogbaya | -42.88 |
| Ivory Coast | FM Chicago | FM281765 | 5.35 | -4.02 | Abidjan | -40.01 |
| Ivory Coast | FM Chicago | FM281764 | 5.35 | -4.02 | Abidjan | -39.50 |
| Ivory Coast | FM Chicago | FM285871 | 9.74 | -5.38 | Bandama. near Tiebila | -49.73 |
| Ivory Coast | FM Chicago | FM277211 | 7.69 | -5.10 | Bouake | -48.10 |
| Ivory Coast | FM Chicago | FM285865 | 8.36 | -4.43 | Dabakala | -29.53 |
| Ivory Coast | FM Chicago | FM285872 | 7.51 | -7.51 | Deoule Waterfall | -47.48 |
| Ivory Coast | FM Chicago | FM285861 | 10.11 | -5.47 | Diaouala | -10.28 |
| Ivory Coast | FM Chicago | FM285870 | 6.73 | -7.35 | Duekoue | -47.50 |
| Ivory Coast | FM Chicago | FM285863 | 9.61 | -6.94 | Mandinani. mts E | -36.68 |
| Ivory Coast | YPM Yale | YPM ORN 077152 | 7.58 | -8.45 | Mount Nimba | -37.15 |
| Ivory Coast | FM Chicago | FM278900 | 6.56 | -5.02 | Toumodi | -14.12 |
| Kenya | YPM Yale | YPM ORN 079564 | -3.48 | 39.77 | 9 mile nnw of Kilifi | -21.17 |
| Kenya | CM Pittsburgh | CM139787 | 1.40 | 35.80 | CHERENGANY HILLS (NORTHERN) | -3.96 |
| Kenya | FM Chicago | FM370049 | 2.20 | 36.91 | Horr Valley | -11.99 |
| Kenya | AMNH New York | AMNH827065 | 1.17 | 35.22 | Kabolet Forest Cherangani Mts. | -45.40 |
| Kenya | CM Pittsburgh | CM145246 | 0.27 | 34.79 | KAKAMEGA FOREST | -35.45 |
| Kenya | WFVZ Camarillo | WFVZ18369 | -1.14 | 36.85 | Kamiti | -38.08 |
| Kenya | CM Pittsburgh | CM139775 | 1.23 | 35.13 | KAPENGURIA (CHERENGANY HILLS) | -15.23 |
| Kenya | WFVZ Camarillo | WFVZ24131 | -1.29 | 36.64 | Karen | -42.67 |
| Kenya | FM Chicago | FM370052 | -3.62 | 39.83 | Kilifi | -24.30 |
| Kenya | CM Pittsburgh | CM139733 | -0.49 | 36.26 | LAKE ELMENTEITA (S SHORE) | -17.63 |
| Kenya | CM Pittsburgh | CM139610 | -0.79 | 36.42 | LAKE NAIVASHA (E SHORE) | -21.20 |
| Kenya | CM Pittsburgh | CM140036 | 2.37 | 34.93 | LOKITANYALA ESCARPMENT | -31.28 |
| Kenya | AMNH New York | AMNH827062 | -1.85 | 40.11 | Makeri. Lower Tana | -35.15 |
| Kenya | CM Pittsburgh | CM148512 | -3.56 | 38.75 | MAUNGU | -33.00 |
| Kenya | CM Pittsburgh | CM146250 | -1.48 | 36.09 | MOSIRO | -18.02 |
| Kenya | FM Chicago | FM370050 | 2.76 | 36.91 | Mt Kulal | -39.24 |
| Kenya | WFVZ Camarillo | WFVZ18342 | -1.80 | 38.43 | Mutha | -35.86 |
| Kenya | YPM Yale | YPM ORN 085536 | -1.28 | 36.83 | Nairobi | -23.00 |
| Kenya | CM Pittsburgh | CM149055 | 0.02 | 37.08 | NANYUKI (NORTHERN EWASO NG'IRO) | -36.72 |
| Kenya | CM Pittsburgh | CM146431 | -1.09 | 35.87 | NAROK | -29.98 |
| Kenya | AMNH New York | AMNH827058 | 1.19 | 37.31 | Ngeng River. Mathews Range | -46.49 |
| Kenya | YPM Yale | YPM ORN 080170 | 0.23 | 37.88 | Nyambeni Mountains | -38.38 |
| Kenya | CM Pittsburgh | CM149394 | -0.66 | 36.83 | NYERI (20 MI SSW) | -59.46 |
| Kenya | CM Pittsburgh | CM149443 | -1.90 | 35.75 | OLMESUTYE (LOITA HILLS) | -44.02 |
| Kenya | WFVZ Camarillo | WFVZ18370 | -1.24 | 36.89 | Ruaraka | -42.63 |
| Kenya | MNHN Paris | MNHN19681147 | 0.26 | 36.54 | Rumuruti area | -3.81 |
| Kenya | CM Pittsburgh | CM147572 | -0.67 | 37.21 | SAGANA | -36.53 |
| Kenya | FM Chicago | FM370046 | 2.10 | 36.92 | South Horr | -24.57 |
| Kenya | CM Pittsburgh | CM149027 | 0.98 | 37.30 | WAMBA | -27.33 |
| Kenya | AMNH New York | AMNH827060 | -2.39 | 40.51 | Witu. Utwani Forest | -32.29 |
| Liberia | AMNH New York | AMNH825531 | 5.53 | -8.23 | Dugbe River ca. 12 mi SSE Jaoudi approx. 5°32'N. 8°14'W | -25.43 |
| Liberia | AMNH New York | AMNH825524 | 5.00 | -9.03 | Greenville. New Kru Town | -38.07 |
| Liberia | AMNH New York | AMNH825530 | 5.42 | -8.82 | Juarzon | -35.48 |
| Liberia | AMNH New York | AMNH827719 | 8.16 | -9.50 | Ziggida 13 Km. N. 1 Km. E. | -33.05 |
| Malawi | YPM Yale | YPM ORN 085538 | -16.51 | 35.16 | Chiromo. Port Herald. Nyasaland | -47.48 |
| Malawi | FM Chicago | FM474885 | -14.43 | 35.46 | Mogochi Hills Forest Reserve | -34.88 |
| Malawi | FM Chicago | FM474875 | -14.01 | 35.32 | Namizimo Forewst Reserve. Kwitunji camp 1 Km SW | -31.84 |
| Malawi | FM Chicago | FM444343 | -13.38 | 34.00 | Ntchisi Forest Reserve | -43.79 |
| Malawi | FM Chicago | FM447631 | -15.97 | 35.66 | Rua River. Mulanje Mountain | -57.92 |
| Malawi | FM Chicago | FM447623 | -15.86 | 35.59 | Tinyade. Mulanje Mountain | -59.83 |
| Mali | MNHN Paris | MNHN19623718 | 12.64 | -8.00 | Bamako | -32.38 |
| Mali | FM Chicago | FM285230 | 16.78 | -3.01 | Tombouctou | 10.56 |
| Mali | AMNH New York | AMNH822588 | 16.78 | -3.01 | Tombouctou | -11.65 |
| Mozambique | FM Chicago | FM283066 | -19.80 | 34.86 | Beira | -36.63 |
| Mozambique | MNHN Paris | MNHN20110222 | -10.74 | 40.21 | Cabo Delgado. Main Camp site. 2km from Nhica de Rovuma | -33.68 |
| Mozambique | CM Pittsburgh | CM148364 | -18.29 | 34.27 | CANGANETOLE | -44.65 |
| Mozambique | FM Chicago | FM283062 | -19.50 | 34.83 | Dondo Forest. 10 mi NE Dondo | -48.57 |
| Mozambique | FM Chicago | FM481427 | -18.67 | 34.01 | Gorongosa Mt. Murombodzi Waterfall Camp | -40.33 |
| Mozambique | FM Chicago | FM481425 | -18.67 | 34.01 | Gorongosa Mt. Murombodzi Waterfall Camp | -40.05 |
| Mozambique | FM Chicago | FM283065 | -19.76 | 34.89 | North Beira. Savane Rd | -23.38 |
| Mozambique | FM Chicago | FM283068 | -19.82 | 34.32 | Tica. 25 mi S. Buzi River | -44.14 |
| Namibia | MNHN Paris | MNHN19791404 | -18.06 | 21.44 | Andara. Okavango river | -54.58 |
| Namibia | FM Chicago | FM220011 | -21.99 | 16.92 | Okahandja | -49.30 |
| Namibia | YPM Yale | YPM ORN 036818 | -19.17 | 15.90 | Okaukuejo | -34.83 |
| Namibia |  | CH03 | -22.55 | 17.09 | Outeniqua Street nº9 Klein-Windhoek | -6.83 |
| Niger | MNHN Paris | MNHN19880742 | 13.51 | 2.11 | Niamey | -41.68 |
| Nigeria | NBC Leiden | RMNH.AVES.76671 | 9.07 | 4.96 | Bacita | -52.72 |
| Nigeria | DMNH Wilmington | DMNH57214 | 10.16 | 4.35 | Borgu National Park. west of Kainji Lake | -33.86 |
| Nigeria | CM Pittsburgh | CM142380 | 7.43 | 3.88 | IBADAN (UNIVERSITY AREA) | -48.20 |
| Nigeria | DMNH Wilmington | DMNH57212 | 7.43 | 3.88 | Ibadan. University of Ibadan | -34.91 |
| Republic of the Congo | MNHN Paris | MNHN19650854 | -4.27 | 15.26 | Brazaville | -65.37 |
| Republic of the Congo | NBC Leiden | RMNH.AVES.92611 | -4.12 | 15.33 | Kimpoko. 04.12 S. 15.33 E. | -37.03 |
| Republic of the Congo | MNHN Paris | MNHN19701183 | -4.20 | 15.43 | M'Bamou | -48.87 |
| Republic of the Congo | MNHN Paris | MNHN19650853 | -3.68 | 13.35 | Sibiti | -72.91 |
| Senegal | MNHN Paris | MNHN19830142 | 14.56 | -17.01 | Bandia | -51.85 |
| Senegal | YPM Yale | YPM ORN 045660 | 14.63 | -17.45 | Dakar | -41.50 |
| Senegal | MNHN Paris | MNHN19840506 | 14.07 | -16.26 | Foret de Oalor | -34.04 |
| Senegal | MNHN Paris | MNHN19680921 | 16.27 | -15.80 | Mbané. 20km Sd Richard to | -65.29 |
| Senegal | MNHN Paris | MNHN19720953 | 14.20 | -16.87 | Ngazobil | -35.32 |
| Sierra Leone | KU Lawrence | KU115018 | 8.02 | -11.43 | Kambui Hills Forest Reserve | -48.88 |
| Sierra Leone | KU Lawrence | KU115336 | 9.68 | -12.18 | Outamba-Kilimi National Park | -45.94 |
| Somalia | YPM Yale | YPM ORN 035219 | 9.93 | 43.18 | Borama | -8.50 |
| Somalia | MNHN Paris | MNHN19641405 | 0.58 | 42.72 | Ile Alessandra | -35.86 |
| Somalia | MNHN Paris | MNHN19641408 | 0.58 | 42.72 | Ile Alessandra | -50.79 |
| South Africa | YPM Yale | YPM ORN 093127 | -33.00 | 27.90 | East London | -19.24 |
| South Africa | YPM Yale | YPM ORN 072426 | -28.63 | 32.07 | Mposa | -32.68 |
| South Africa | YPM Yale | YPM ORN 040644 | -29.87 | 30.93 | near Durban | -37.37 |
| South Africa | YPM Yale | YPM ORN 069089 | -30.32 | 30.74 | Park Rynie | -25.51 |
| South Africa | YPM Yale | YPM ORN 069297 | -28.57 | 32.11 | Penicuik | -19.97 |
| South Africa | YPM Yale | YPM ORN 085539 | -29.33 | 31.30 | Stanger | -24.97 |
| South Africa | MNHN Paris | MNHN19810154 | -31.56 | 29.62 | Transkei Coast- Lusikisiki district. Ntafufu river | -42.13 |
| South Africa | MNHN Paris | MNHN19810153 | -31.56 | 29.62 | Transkei Coast- Lusikisiki district. Ntafufu river | -33.20 |
| South Africa | MNHN Paris | MNHN19720296 | -28.67 | 29.97 | Tugela river. 10 miles au NE de Colenso Kwzulunatal | -27.80 |
| South Africa | FM Chicago | FM279202 | -29.58 | 30.27 | Winterskloof | -36.48 |
| Sudan | FM Chicago | FM103311 | 4.41 | 32.56 | Torit | -10.38 |
| Swaziland | MNHN Paris | MNHN19692883 | -26.08 | 31.52 | Komati river. near Baligane | -30.52 |
| Swaziland | MNHN Paris | MNHN19692884 | -26.08 | 31.52 | Komati river. near Baligane | -34.24 |
| Tanzania | YPM Yale | YPM ORN 093106 | -6.73 | 39.22 | 9 miles n of Dar es Salaam | -32.45 |
| Tanzania | CM Pittsburgh | CM147136 | -5.09 | 38.60 | AMANI (USAMBARA MOUNTAINS) | -46.25 |
| Tanzania | DMNH Wilmington | DMNH81929 | -5.94 | 39.27 | Chaani | -40.69 |
| Tanzania | YPM Yale | YPM ORN 093111 | -8.87 | 34.03 | Chimala | -67.48 |
| Tanzania | CM Pittsburgh | CM146766 | -5.41 | 35.82 | DODOMA (50 MI N) | -35.14 |
| Tanzania | YPM Yale | YPM ORN 093098 | -7.82 | 35.65 | Iringa | -39.84 |
| Tanzania | AMNH New York | AMNH827056 | -6.84 | 37.59 | Kimbora Forest | -36.33 |
| Tanzania | YPM Yale | YPM ORN 093133 | -4.51 | 38.39 | Lunguza | -25.62 |
| Tanzania | NHMD | 100627 | -6.20 | 29.83 | Mahale mountains NP | -24.66 |
| Tanzania | NBC Leiden | RMNH.AVES.36665 | -10.28 | 40.12 | Mikindani | -50.91 |
| Tanzania | DMNH Wilmington | DMNH81930 | -6.07 | 39.25 | Mpapa | -51.37 |
| Tanzania | YPM Yale | YPM ORN 093113 | -3.25 | 36.73 | near Usa River | -46.80 |
| Tanzania | YPM Yale | YPM ORN 093103 | -6.85 | 39.30 | Pugu Hills s of Dar es Salaam | -45.15 |
| Tanzania | YPM Yale | YPM ORN 093135 | -4.08 | 37.72 | Same | -29.01 |
| Tanzania | NBC Leiden | RMNH.AVES.37613 | -6.83 | 38.88 | Soga | -28.40 |
| Tanzania | YPM Yale | YPM ORN 093124 | -6.82 | 37.67 | Uluguru Mountains near Morogoro | -34.33 |
| Tanzania | YPM Yale | YPM ORN 093142 | -3.67 | 35.83 | west side of Lake Manyara | -51.31 |
| Tchad | MNHN Paris | MNHN20091029 | 7.90 | 16.04 | Bekao | -38.17 |
| Tchad | MNHN Paris | MNHN19780509 | 7.66 | 15.92 | Dagbao | -13.21 |
| Tchad | MNHN Paris | MNHN19790563 | 7.66 | 15.92 | Dagbao | -30.72 |
| Tchad | MNHN Paris | MNHN19560316 | 12.07 | 15.04 | Fort Foureau | -15.78 |
| Tchad | MNHN Paris | MNHN19790564 | 8.42 | 16.16 | Kaga | -40.04 |
| Tchad | MNHN Paris | MNHN19790561 | 8.57 | 16.08 | Moundou | -20.18 |
| Togo | MNHN Paris | MNHN19660604 | 9.55 | 1.19 | Kara | -50.47 |
| Togo | MNHN Paris | MNHN19660603 | 9.55 | 1.19 | Kara | -44.25 |
| Uganda | FM Chicago | FM385145 | -1.03 | 29.62 | Nteko | -30.95 |
| Uganda | AMNH New York | AMNH827063 | 0.81 | 30.09 | Ntotoro. Bwamba Forest | -57.69 |
| Uganda | FM Chicago | FM346482 | -0.31 | 32.26 | Sese Is. Bugala Id. Kalangala | -42.85 |
| Zambia | FM Chicago | FM263486 | -14.50 | 22.50 | Liuwa Plain | -69.30 |
| Zambia | FM Chicago | FM263493 | -14.85 | 22.07 | Luachi River | -63.20 |
| Zambia | YPM Yale | YPM ORN 093101 | -8.85 | 31.37 | Mbala | -60.94 |
| Zambia | FM Chicago | FM263489 | -15.58 | 22.00 | S Lueti River. Angola border | -52.70 |
| Zambia | FM Chicago | FM263492 | -15.03 | 22.17 | Sikongo | -49.23 |
| Zimbabwe | MNHN Paris | MNHN19900847 | -16.22 | 30.10 | Angwa river | -40.22 |
| Zimbabwe | MNHN Paris | MNHN19900845 | -16.09 | 30.70 | Zarabani | -38.41 |
